# ICTV Virus Taxonomy Profile: *Paramyxoviridae*

**DOI:** 10.1099/jgv.0.001328

**Published:** 2019-10-14

**Authors:** Bert Rima, Anne Balkema-Buschmann, William G. Dundon, Paul Duprex, Andrew Easton, Ron Fouchier, Gael Kurath, Robert Lamb, Benhur Lee, Paul Rota, Linfa Wang, ICTV Report Consortium

**Affiliations:** ^1^​ Centre for Experimental Medicine, School of Medicine, Dentistry and Biomedical Sciences, The Queen's University of Belfast, Belfast, Northern Ireland, UK; ^2^​ Federal Research Institute for Animal Health, Greifswald – Insel Riems, Germany; ^3^​ Animal Production and Health Laboratory, International Atomic Energy Agency Laboratories Seibersdorf, 2444 Seibersdorf, Austria; ^4^​ Center for Vaccine Research, University of Pittsburgh, PA, USA; ^5^​ School of Life Sciences, University of Warwick, Coventry, UK; ^6^​ Department of Viroscience, Erasmus Medical Center, Rotterdam, The Netherlands; ^7^​ US Geological Survey Western Fisheries Research Center, Seattle, WA, USA; ^8^​ Department of Molecular Biosciences and Howard Hughes Medical Institute, Northwestern University, Evanston, IL, USA; ^9^​ Icahn School of Medicine at Mount Sinai, NY, USA; ^10^​ National Center for Immunization and Respiratory Diseases, Centers for Disease Control and Prevention, Atlanta, GA, USA; ^11^​ Program in Emerging Infectious Diseases, Duke-NUS Graduate Medical School, 8 College Rd, Singapore

**Keywords:** ICTV Report, Taxonomy, *Paramyxoviridae*

## Abstract

The family *Paramyxoviridae* consists of large enveloped RNA viruses infecting mammals, birds, reptiles and fish. Many paramyxoviruses are host-specific and several, such as measles virus, mumps virus, Nipah virus, Hendra virus and several parainfluenza viruses, are pathogenic for humans. The transmission of paramyxoviruses is horizontal, mainly through airborne routes; no vectors are known. This is a summary of the current International Committee on Taxonomy of Viruses (ICTV) Report on the family *Paramyxoviridae*. which is available at ictv.global/report/paramyxoviridae.

## Virion

**Fig. 1.**
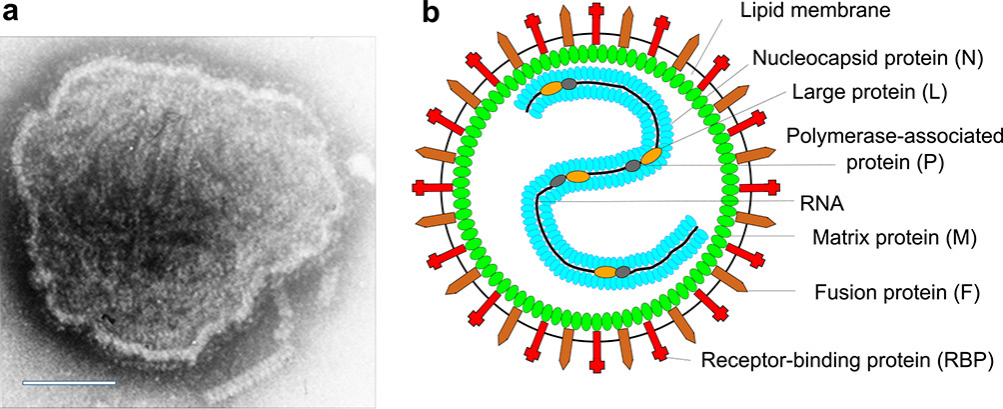
Paramyxovirus virion structure. (a) Negative-contrast electron micrograph of intact measles virus particle (genus *Morbillivirus*). Bar: 100 nm. (b) Schematic diagram of paramyxovirus particle in cross-section.

Virions are enveloped, pleomorphic, but probably mostly spherical (Table 1, Fig. 1), with a ribonucleoprotein (RNP) core containing the RNA genome protected by a helical nucleocapsid protein (N), the polymerase-associated protein (P) and the large protein (L, including RNA-directed RNA polymerase, capping and cap methylation activities). The envelope contains two glycoproteins with receptor attachment [receptor-binding protein (RBP), designated variably as haemagglutinin–neuraminidase protein (HN), haemagglutinin (H) or glycoprotein (G)] and fusion (F) functions.

**Table 1.** Characteristics of members of the family *Paramyxoviridae*

Typical member:	measles virus, Ichinose-B95a (AB016162), species *Measles morbillivirus*, genus *Morbillivirus*
Virion	Enveloped, pleomorphic (mostly spherical) virions with a diameter of 300–500 nm enclosing a ribonucleoprotein
Genome	Negative-sense, non-segmented RNA genomes of 14.6 to 20.1 kb
Replication	Cytoplasmic; the virus ribonucleoprotein complex replicates the antigenome and transcribes 6–8 positive-sense mRNAs
Translation	Cytoplasmic, by cellular machinery from capped and poly-adenylated mRNAs
Host range	Mammals, birds, fish and reptiles
Taxonomy	Realm *Riboviria*, phylum *Negarnaviricota*, class *Monjiviricetes*, order *Mononegavirales*. Several subfamilies, numerous genera and >70 species

## Genome

**Fig. 2.**
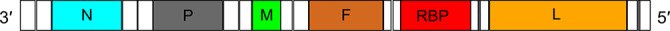
Paramyxovirus genome structure (not to scale). Open reading frames (ORFs) are labelled as in Figure 1. Non-coloured regions represent untranslated regions in the mRNAs.

Virus genomes range from 14 296–20 148 nt, but all have a canonical gene arrangement of: 3′-N-P/V/C-M-F-RBP-L-5′ (Fig. 2). In some members this is interspersed with additional transcription units (ATUs).

## Replication

Transcription of the negative-sense genome occurs in the cytoplasm and starts with the binding of the P/L protein complex to the transcription promoter at the 3′-end of the RNA, and transcription of 6–8 mRNAs that are capped and poly-adenylated. N-co-terminal V and P proteins encoded by alternative reading frames in the second transcribed gene are accessed by co-transcriptional insertion of non-templated G residues. The C protein is encoded in an overlapping reading frame in the V/P mRNA of most paramyxoviruses, with the exception of members of the subfamilies *Avulavirinae*, *Rubulavirinae, Metaparamyxovirinae* and the genus *Ferlavirus*. During replication, the negative-sense ribonucleoprotein (RNP) template is copied into a full-length encapsidated positive-sense RNA. Negative-sense RNPs are transported to the cellular surface, where budding occurs through interaction of the matrix (or membrane, M) protein with the RNP and the cytoplasmic tails of the two glycoproteins: the fusion protein (F) and the attachment protein (RBP).

## Taxonomy

Subfamilies are defined by phylogenetic analysis of the L protein [1, 2], and are consistent with previous classifications based on host range, biological or biochemical criteria [3–5].

### *Avulavirinae*

Members of the genera *Orthoavulavirus, Metaavulavirus* and *Paraavulavirus* infect birds. Their RBP has haemagglutinin and neuraminidase activity. Their genomes lack ATUs.

### *Rubulavirinae*

Many members of the genera *Orthoarubulavirus* and *Pararubulavirus* derive from bats. Important human viruses are mumps virus and the respiratory viruses human parainfluenzavirus 2 and 4. The RBP of orthorubulaviruses has both haemagglutinin and neuraminidase activity. Pararubulavirus RBPs probably lack neuraminidase activity and may use a receptor other than sialic acid. Mumps virus and parainfluenza virus 5 have an ATU encoding an SH protein between the F and RBP genes.

### *Orthoparamyxovirinae*

Members of the genera *Respirovirus*, *Aquaparamyxovirus*, *Henipavirus*, *Narmovirus*, *Morbillivirus* and *Salemvirus* lack ATUs. Members of the genus *Ferlavirus* have an additional ATU (U) between the N and P genes encoding a protein with unknown function. Members of *Jeilongvirus* have one or two ATUs (encoding SH and/or tM proteins) between the F and RBP genes. Members of the genera *Respirovirus*, *Aquaparamyxovirus*, *Ferlavirus* and possibly *Jeilongvirus* have a RBP that possesses haemagglutinin and neuraminidase activities. Members of the genera *Henipavirus* and *Morbillivirus* (and probably *Narmovirus* and *Salemvirus*) have protein receptors.

### *Metaparamyxovirinae*

The species *Synodus synodonvirus* has been established based on two sequences found in a triplecross lizardfish. The RBP of Wēnlǐng triplecross lizardfish paramyxovirus probably interacts with a protein receptor.

## Resources

Current ICTV Report on the family *Paramyxoviridae*: ictv.global/report/paramxyoviridae.
